# Predictors of WIC uptake among low-income pregnant individuals: a longitudinal nationwide analysis

**DOI:** 10.1016/j.ajcnut.2023.04.023

**Published:** 2023-04-23

**Authors:** Daniel F. Collin, Alice Guan, Rita Hamad

**Affiliations:** 1 Philip R. Lee Institute for Health Policy Studies, University of California San Francisco, San Francisco, CA, United States; 2 Department of Epidemiology and Biostatistics, University of California San Francisco, San Francisco, CA, United States; 3 Department of Family and Community Medicine, University of California San Francisco, San Francisco, CA, United States

**Keywords:** nutrition, pregnancy, health disparities, WIC uptake, PRAMS

## Abstract

**Background:**

Nutrition during pregnancy is important for maternal and infant health. The Special Supplemental Nutrition Program for Women, Infants, and Children (WIC) provides nutritional support for low-income pregnant and postpartum individuals and children under the age of 5 y. However, WIC participation was in decline in the decade leading up to 2019.

**Objectives:**

This study examined individual and state predictors associated with WIC uptake among eligible individuals so as to identify subgroups for targeted intervention to improve participation.

**Methods:**

Data came from the 2004–2019 waves of the Pregnancy Risk Assessment Monitoring System (PRAMS), a national survey of individuals who recently gave birth (*N* = 288,531). Multivariable logistic regressions were used to examine individual- and state-level and temporal predictors of WIC uptake among WIC-eligible respondents.

**Results:**

Among WIC-eligible respondents, ages of >35 (OR: 0.68; 95% CI: 0.66, 0.70), more than high school education (OR: 0.63; 95% CI: 062, 0.65), English language proficiency (OR: 0.71; 95% CI: 0.68, 0.74), being married (OR: 0.70; 95% CI: 0.69, 0.72), White race, smaller family size, not having prepregnancy diabetes, and higher income were associated with lower odds of WIC uptake. Respondents in states with higher earned income tax credit rates and in the Northeast, Midwest, and West (compared with the South) had lower WIC uptake. Respondents in states with higher gross domestic product, higher unemployment rates, higher Supplemental Nutrition Assistance Program, Temporary Assistance for Needy Families, and Medicaid caseloads, and Democrat governors had higher uptake; however, effect estimates were small and may not represent a meaningful change. Associations were the strongest during 2009–2015 than during other years, particularly for race/Hispanic origin, language, marital status, prepregnancy diabetes, family size, and prepregnancy.

**Conclusions:**

This study identified several individual- and state-level characteristics associated with WIC uptake among low-income eligible respondents, paving the way for future interventions to target key subgroups to improve program participation.

## Introduction

Poor nutrition during pregnancy is linked to adverse maternal health during the perinatal period and beyond, as well as worsened infant outcomes (e.g., low birthweight and failing to meet developmental milestones) that can persist during childhood and throughout the life course [1,2]. Socioeconomic and racial disparities in maternal nutrition, particularly among Black and Hispanic mothers and those with low income, can lead to worsened health among these groups [3]. In the United States, safety net programs such as the Special Supplemental Nutrition Program for Women, Infants, and Children (WIC) were designed to safeguard the health of low-income pregnant individuals and children. WIC is the third largest nutrition assistance program in the United States and provides food, nutrition education, breastfeeding support, and referrals to health care and social services for low-income pregnant and postpartum persons and children under the age of 5 y [4,5].

WIC participation has been associated with several maternal and infant health benefits, including improved diet quality during pregnancy and reduced food insecurity [6,7]. Program revisions in 2009, which aligned WIC food packages with the U.S. Department of Agriculture (USDA) Dietary Guidelines for Americans, have also been found to reduce the risk of preeclampsia and increase the likelihood of gaining the recommended amount of weight during pregnancy among participating adults [8]. Among infants, participation has been linked to improved birthweight, and reduced preterm birth and neonatal and fetal mortality [[9], [10], [11]]. Improvements tend to be stronger among Black and Hispanic participating adults and infants [12,13].

Despite the benefits associated with WIC participation, program participation among eligible individuals remains low and was decreasing through 2019. In 2019, 11 million people were eligible for WIC each month, yet only 6.7 million people participated in the program, indicating an uptake rate of 57% compared with 62% in 2010 [14]. The uptake was lower for pregnant (52%) compared with postpartum (85%) individuals; among children, the uptake rate was lowest for 4-y olds (25%) and highest for infants (98%). The uptake is highest among Hispanic (67%) individuals, compared with those of Black (62%) and White (46%) individuals. While WIC is a national program, there is also variation in uptake by state, with Vermont having the highest rate (73%) and 12 states having rates of <50%. Safety net programs such as WIC may buffer against detrimental effects during periods of economic instability, as observed by the increased participation during the Great Recession of 2007–2009 and the COVID-19 pandemic [[15], [16], [17], [18]].

Given the benefits associated with WIC participation, it is important to examine the factors associated with low participation. The current study aims to examine individual- and state-level predictors associated with WIC uptake and how these associations changed during periods of economic instability. The study used the most recently available data from a large national longitudinal survey. Findings may help inform outreach and targeting efforts to improve WIC uptake.

## Methods

### Data

This study used data from the Pregnancy Risk Assessment Monitoring System (PRAMS), a surveillance project of the U.S. Centers for Disease Control and Prevention in conjunction with participating sites (state, territorial, or local health departments). PRAMS includes a representative sample of individuals who had a live birth and collects survey responses on sociodemographic information and health before, during, and shortly after pregnancy. These data are then linked with additional variables from birth certificates. Each participating site samples between 1300 and 3400 individuals per year. Several states do not participate in this federal data collection effort, so PRAMS represents approximately 81% of all live births in USA. In addition, PRAMS makes data available only for sites that meet a minimum response rate threshold in a given year; this ranged from 50% to 70% during the study period. More detailed methodology on PRAMS has been previously described [19].

### Sample selection

**FIGURE 1:**
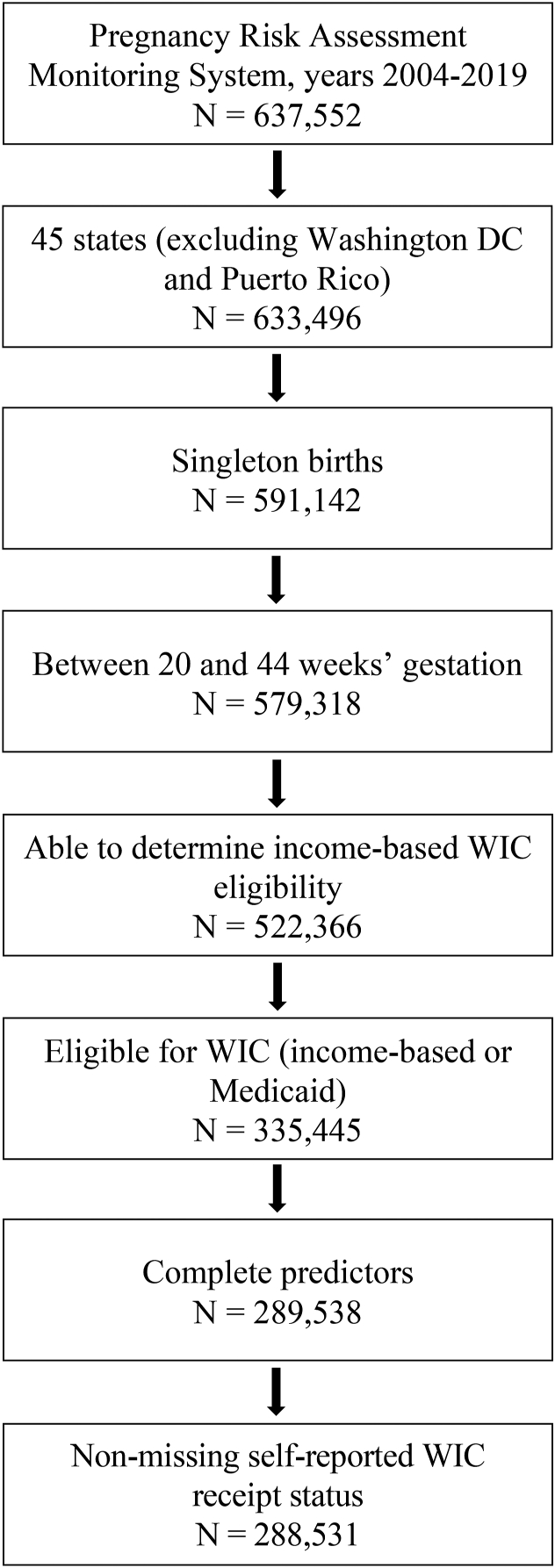
Sample selection flowchart. The sample was drawn from PRAMS participating sites from 2004 to 2019 and included respondents with live-born singleton births with a gestational age of 20–44 wk at delivery, who were eligible for WIC (based on income threshold or Medicaid receipt during pregnancy). PRAMS, Pregnancy Risk Assessment Monitoring System; WIC, Special Supplemental Nutrition Program for Women, Infants, and Children.

This study used PRAMS survey waves 2004–2019 (*N* = 637,552). Data prior to 2004 were excluded due to differences in how birth certificate data were collected, and 2019 was the most recent year of data available at the start of the analyses. This study included respondents with live-born singleton births with a gestational age of 20–44 wk at delivery from 45 states for which PRAMS makes data available (Figure 1; **Supplemental Table**). The sample was then restricted to respondents for whom information on income and family size was nonmissing (*N* = 522,366), in order to determine income-based WIC eligibility using the USDA’s annual guidelines (i.e., below 185% of the federal poverty level for family size). The sample was further restricted to the analytic sample of those who were WIC-eligible, imputed based on each respondent’s self-reported income the year before delivery, family size, and state of residence, or if they reported having Medicaid for prenatal care (since Medicaid confers adjunctive eligibility; *N* = 335,445). Of note, PRAMS reports income as a categorical variable using different income ranges in different years. Therefore, this study used the maximum income value in each income category in each year to impute an individual’s WIC eligibility. This means that the sample was likely to exclude some eligible individuals, e.g., if their income fell in the bottom of a particular category’s range and if that range included that year’s WIC eligibility threshold. However, this reduced the chances that the sample included higher-income ineligible respondents. The sample was then restricted to those with complete predictors (*N* = 289,538) and further restricted to those with information on WIC receipt during pregnancy (*N* = 288,531).

## Outcome

The primary outcome was a dichotomous variable indicating whether a respondent self-reported WIC receipt during pregnancy in either the PRAMS survey or the linked birth certificate.

### Explanatory variables

Individual-level predictors included a respondent’s age, race/Hispanic origin, education, family size, household income, language, prepregnancy diabetes, pregnancy intent, and census region of residence. Race and Hispanic origin were self-reported as 2 separate questions in the linked birth certificate data and were categorized in this study as non-Hispanic White, non-Hispanic Black, Hispanic, non-Hispanic Asian/Pacific Islander, non-Hispanic American Indian/Alaska Native, and non-Hispanic other. Pregnancy intent was coded as a binary variable (wanted to get pregnant for the pregnancy in question; compared with those who wanted to get pregnant sooner, later, or not at all). Additional details on covariate categories are included in the Supplementary material.

In addition, while WIC is a federal program, it is administered by state agencies, which results in some state-level variation in enrollment practices. Therefore, this study included yearly state-level predictors: GDP per capita, unemployment rate, state earned income tax credit (EITC) rate, an indicator for whether the governor was a Democrat, and measures of state caseloads of other social programs including the Supplemental Nutrition Assistance Program (SNAP), Temporary Assistance for Needy Families program (TANF), and Medicaid. Additional details are included in the Supplementary material.

### Analysis

The study first tabulated individual- and state-level characteristics for the full sample of WIC-eligible respondents. Then, the study compared whether there were important differences across groups by calculating standardized differences, a statistic that is not influenced by the large sample size. This involves taking the difference between the 2 groups and dividing this difference by the standard deviation. Unadjusted logistic regression models were used to examine the bivariate association between each individual- and state-level predictor and WIC uptake. Multivariable logistic regression, adjusting for all individual- and state-level predictors in the model, was then used to examine the association of these predictors with self-reported WIC receipt during pregnancy. Indicator variables for year of delivery (i.e., year fixed effects) were also included in the model to account for underlying secular trends in the outcome that affected all individuals similarly.

To examine whether the predictors for WIC uptake differed over time due to economic or political conditions, the analysis was conducted separately for the periods before and after the Great Recession (2004–2008 compared with 2009–2015; when uptake began to decline more substantially) and after the 2016 presidential election (2016–2019), a period of uncertainty about funding for safety net programs and plans to expand the “public charge” rule [20,21]. Given that states may vary in how they administer WIC services, the main analysis was repeated including state of residence (instead of region) as indicator variables to further examine state-specific coefficients. For this analysis, Illinois was set as the reference state because it is a large state with demographics similar to those of the United States as a whole and with approximately average WIC uptake. Additional sensitivity analyses are included in the Supplementary material.

### Ethics approval

Ethical approval for this study was provided by the Institutional Review Board of the senior author’s university (18-26719).

## Results

### Sample characteristics

Approximately 74% of the WIC-eligible respondents in the study sample reported receiving WIC during pregnancy (Table 1). Nearly half (45.8%) were aged 25–34 y. About 40.4% were White, 23.1% were Black, 21.8% were Hispanic, 5.5% were Asian/Pacific Islander, 5.2% were American Indian/Alaska Native, and 3.9% were other races. About 22.9% had less than high school education, and 40.6% were married. The majority (89.5%) completed the PRAMS survey in English compared with 10.5% respondents who completed in Spanish. About 3.3% had prepregnancy diabetes, and 32.8% intended to get pregnant for the pregnancy in question. The highest proportion (31.0%) of respondents resided in the South census region. Analysis of standardized differences showed differences for age, race/Hispanic origin, education, marital status, and income, indicating differences in these characteristics between WIC recipients and nonrecipients. State characteristics were similar between WIC recipients and nonrecipients, with the only substantive difference for the unemployment rate.

**TABLE 1:** Sample characteristics

	Full sample	Eligible Non-recipient	Eligible WIC recipient	Standardized difference	Uptake rate (%)
	*N* = 288,531	*N* = 75,241	*N* = 213,290		
	% or Mean (SD)	% or Mean (SD)	% or Mean (SD)		
*Individual Characteristics*					
Age (years)					
<25	43.7	34.5	47.0	0.26	79.4
25–34	45.8	52.2	43.5	0.17	70.3
35+	10.5	13.3	9.5	0.12	67.0
Race/Hispanic-origin					
White	40.4	51.0	36.7	0.29	67.1
Black	23.1	16.9	25.3	0.21	80.9
Hispanic	21.8	16.5	23.7	0.18	80.3
Asian/Pacific Islander	5.5	7.3	4.9	0.10	65.4
American Indian/Alaska Native	5.2	4.2	5.6	0.07	79.2
Other	3.9	4.2	3.8	0.02	72.3
Education					
Less than high school	22.9	15.2	25.6	0.26	82.7
High school	37.6	33.0	39.3	0.13	77.2
More than high school	39.4	51.8	35.1	0.34	65.7
Married					
No	59.4	46.9	63.7	0.34	79.4
Yes	40.6	53.1	36.3		66.0
Family income^1^, ^2^ ≥ $50,000 USD					
No	92.4	86.9	94.3	0.25	75.5
Yes	7.6	13.1	5.7		55.4
Family size, including unborn infant^2^					
2	13.3	10.8	14.2	0.10	78.8
3	28.6	27.6	29.0	0.03	74.8
4	26.2	27.5	25.8	0.04	72.6
5	16.8	17.5	16.6	0.03	72.8
6+	15.1	16.5	14.5	0.05	71.4
Language					
Spanish	10.5	6.4	11.9	0.19	84.2
English	89.5	93.6	88.1		72.3
Pre-pregnancy diabetes					
No	96.7	97.2	96.5	0.04	73.8
Yes	3.3	2.8	3.5		78.2
Intended to get pregnant					
No	67.2	64.4	68.2	0.08	75.0
Yes	32.8	35.6	31.8		71.7
Census region					
South	31.0	26.6	32.5	0.13	77.6
Northeast	18.4	17.7	18.6	0.02	74.8
Midwest	24.3	24.2	24.4	0.01	74.1
West	26.3	31.5	24.5	0.16	68.8
WIC receipt during pregnancy	73.9	0.0	100.0		
*State Characteristics*					
GDP per capita (thousands of USD)	49.4 (11.8)	50.7 (11.7)	49.0 (11.8)	0.15	-
Unemployment rate	5.8 (1.9)	5.5 (1.8)	5.9 (1.9)	0.23	-
EITC rate	7.3 (9.8)	7.1 (9.5)	7.4 (9.9)	0.02	-
SNAP caseload	5.6 (2.2)	5.5 (2.2)	5.7 (2.1)	0.09	-
TANF caseload	0.5 (0.2)	0.5 (0.3)	0.5 (0.2)	0.10	-
Medicaid caseload	18.0 (5.9)	17.9 (6.3)	18.1 (5.7)	0.03	-
Governor Democrat	47.9	44.8	49.0	0.09	-

Note: Sample was drawn from PRAMS participating sites from 2004–2019 and included respondents with live-born singleton births with a gestational age of 20–44 weeks at delivery and were eligible for WIC (based on income threshold or Medicaid receipt during pregnancy).

Abbreviations: EITC, Earned Income Tax Credit; GDP, Gross Domestic Product; PRAMS, Pregnancy Risk Assessment Monitoring System; SNAP, Supplemental Nutrition Assistance Program; TANF, Temporary Assistance for Needy Families; WIC, Special Supplemental Nutrition Program for Women, Infants, and Children.

1 Inflation adjusted to 2018 US dollars; based on the maximum income value in the PRAMS income category to which each individual was assigned.

2 Family size and income were included as a continuous variable in the main analyses, but as a categorical variable in this analysis, to allow for better comparability with the 2019 USDA WIC report. (22).

### Predictors of WIC uptake: bivariate analysis

Bivariate analysis showed that WIC uptake varied by demographic characteristics, with higher uptake among respondents who were younger, Black or Hispanic, with low educational attainment, unmarried, lower income, having one child, Spanish speakers, had prepregnancy diabetes, did not intend to get pregnant, and among those in the South (Table 2). Bivariate analysis also showed that state-level predictors were associated with WIC uptake.

**TABLE 2:** Individual- and state-level predictors of WIC uptake rate, bivariate and multiple imputation analysis

	Bivariate analysis		Multiple imputation	
	OR	95% CI	OR	95% CI
*Individual Predictors*				
Age (years)				
<25 (Reference)				
25–34	0.61∗∗	(0.60, 0.62)	0.79∗∗	(0.77, 0.80)
35+	0.53∗∗	(0.51, 0.54)	0.68∗∗	(0.66, 0.70)
Race/Hispanic-origin				
White (Reference)				
Black	2.08∗∗	(2.03, 2.13)	1.77∗∗	(1.72, 1.81)
Hispanic	2.00∗∗	(1.96, 2.05)	1.64∗∗	(1.6, 1.69)
Asian/Pacific Islander	0.93∗∗	(0.89, 0.96)	1.17∗∗	(1.13, 1.21)
American Indian/Alaska Native	1.86∗∗	(1.79, 1.94)	1.83∗∗	(1.76, 1.91)
Other	1.28∗∗	(1.23, 1.34)	1.42∗∗	(1.36, 1.48)
Education				
Less than high school (Reference)				
High school	0.71∗∗	(0.69, 0.72)	0.89∗∗	(0.86, 0.91)
More than high school	0.40∗∗	(0.39, 0.41)	0.63∗∗	(0.61, 0.64)
Married				
No (Reference)				
Yes	0.50∗∗	(0.49, 0.51)	0.71∗∗	(0.70, 0.72)
Family income^1^, ^2^ ≥ $50,000 USD				
No (Reference)				
Yes	0.40∗∗	(0.39, 0.42)	0.57∗∗	(0.55, 0.58)
Family size, including unborn infant^2^				
2 (Reference)				
3	0.80∗∗	(0.78, 0.82)	0.96∗∗	(0.93, 0.99)
4	0.71∗∗	(0.69, 0.73)	0.93∗∗	(0.90, 0.95)
5	0.72∗∗	(0.70, 0.74)	0.99	(0.96, 1.02)
6+	0.67∗∗	(0.65, 0.69)	1.02	(0.99, 1.06)
Language				
Spanish (Reference)				
English	0.50∗∗	(0.49, 0.52)	0.7∗∗	(0.68, 0.73)
Pre-pregnancy diabetes				
No (Reference)				
Yes	1.28∗∗	(1.21, 1.34)	1.28∗∗	(1.22, 1.34)
Intended to get pregnant				
No (Reference)				
Yes	0.84∗∗	(0.83, 0.86)	0.99	(0.98, 1.01)
Census region of residence				
South (Reference)				
Northeast	0.86∗∗	(0.83, 0.88)	0.90∗∗	(0.87, 0.93)
Midwest	0.82∗∗	(0.80, 0.84)	0.90∗∗	(0.87, 0.92)
West	0.63∗∗	(0.62, 0.65)	0.69∗∗	(0.67, 0.71)
*State Predictors*				
GDP per capita (thousands of USD)	0.988∗∗	(0.987, 0.989)	1.004∗∗	(1.002, 1.005)
Unemployment rate	1.13∗∗	(1.127, 1.137)	1.02∗∗	(1.01, 1.03)
EITC rate	1.003∗∗	(1.0017, 1.0034)	0.998∗∗	(0.997, 0.999)
SNAP caseload	1.044∗∗	(1.040, 1.048)	1.06∗∗	(1.05, 1.07)
TANF caseload	1.50∗∗	(1.45, 1.55)	1.09∗∗	(1.04, 1.14)
Medicaid caseload	1.006∗∗	(1.004, 1.007)	1.008∗∗	(1.006, 1.010)
Governor Democrat	1.0017∗∗	(1.0015, 1.0019)	1.0004∗∗	(1.0002, 1.0006)

∗ *P* < 0.05, ∗∗ *P* < 0.01.

Note: Sample (N=288,531) was drawn from PRAMS participating sites from 2004–2019 and included respondents with live-born singleton births with a gestational age of 20–44 weeks at delivery and were eligible for WIC (based on income threshold or Medicaid receipt during pregnancy). Bivariate analyses involved unadjusted logistic regression analysis examining the association between each predictor and WIC uptake. Multiple imputation models involved imputation of missing individual-level covariates using multiple imputation using chained equations (10 imputations), followed by logistic regressions adjusting for individual-level and state-level covariates and year of delivery.

Abbreviations: EITC, Earned Income Tax Credit; GDP, Gross Domestic Product; PRAMS, Pregnancy Risk Assessment Monitoring System; SNAP, Supplemental Nutrition Assistance Program; TANF, Temporary Assistance for Needy Families; USDA, US Department of Agriculture; WIC, Special Supplemental Nutrition Program for Women, Infants, and Children.

1 Inflation adjusted to 2018 US dollars based on the maximum income value in each income category.

2 Family size and income were included as a continuous variable in the main analyses, but as a categorical variable in this analysis, to allow for better comparability with the 2019 USDA WIC report. (22).

### Predictors of WIC uptake: multivariable analysis

**FIGURE 2:**
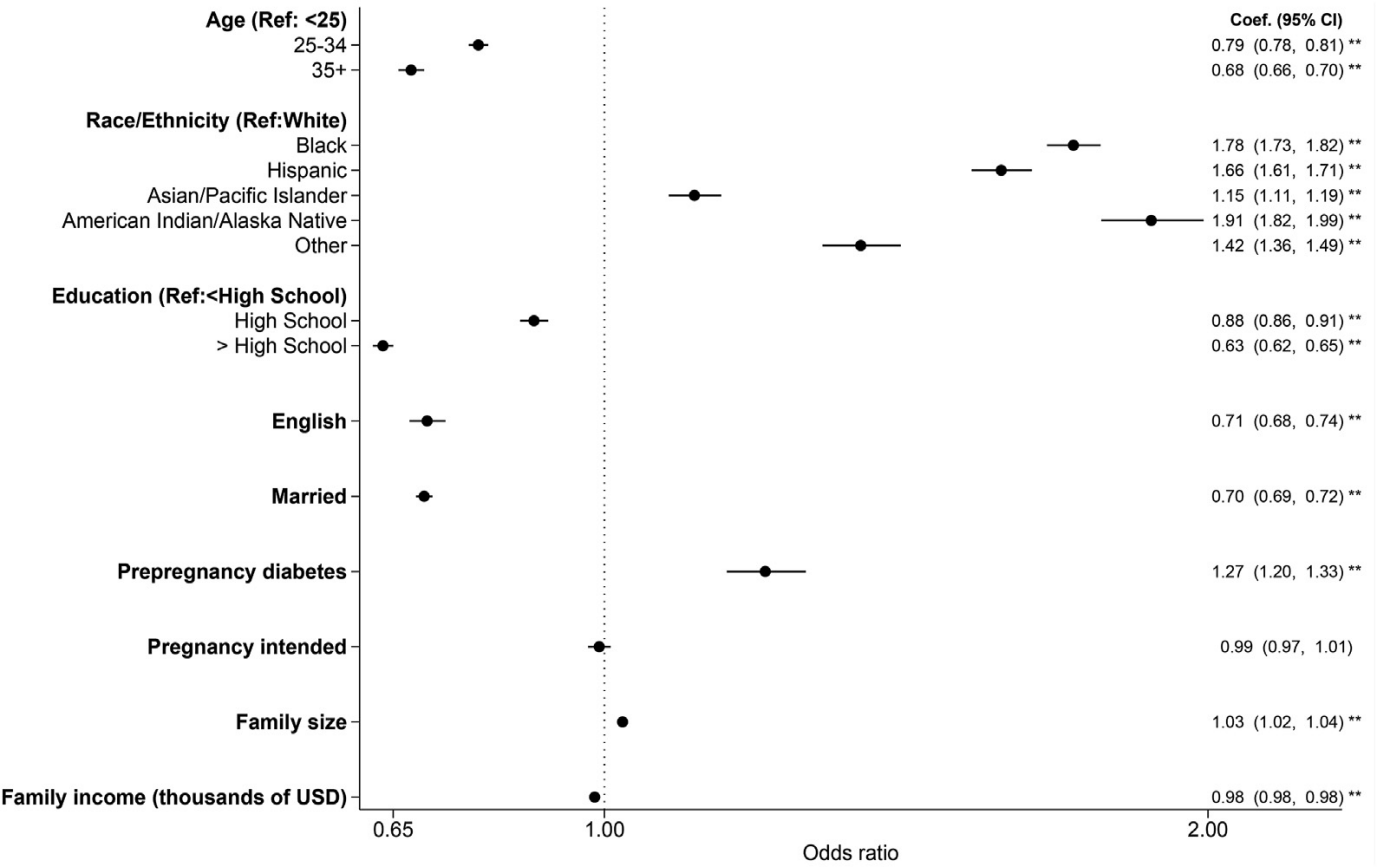
Individual-level predictors of WIC uptake. Sample (*N* = 288,531) was drawn from PRAMS participating sites from 2004 to 2019 and included respondents with live-born singleton births with a gestational age of 20–44 wk at delivery, who were eligible for WIC (based on income threshold or Medicaid receipt during pregnancy). Multivariable logistic regressions examined the association between each predictor and WIC uptake, controlling for individual- and state-level covariates (GDP per capita, unemployment rate, EITC rate, SNAP, TANF, and Medicaid caseloads, governor Democrat, and census region) and the year of delivery. The filled circles and lines indicate ORs and 95% CIs, respectively. ∗∗*P* < 0.01. EITC, earned income tax credit; PRAMS, Pregnancy Risk Assessment Monitoring System; SNAP, Supplemental Nutrition Assistance Program; TANF, Temporary Assistance for Needy Families; WIC, Special Supplemental Nutrition Program for Women, Infants, and Children.

Multivariable models were then used to examine individual- and state-level predictors of WIC uptake among eligible respondents. All these variables were included in the same model but are presented in 2 figures for ease of interpretation. For individual-level predictors (Figure 2), older age, higher education, proficiency in English language, being married, and higher income were all associated with decreased WIC uptake. Black, Hispanic, Asian/Pacific Islander, American Indian/Alaska Native, and respondents of other races had higher uptake compared with White respondents. Having prepregnancy diabetes and larger family size were also associated with higher uptake.

**FIGURE 3:**
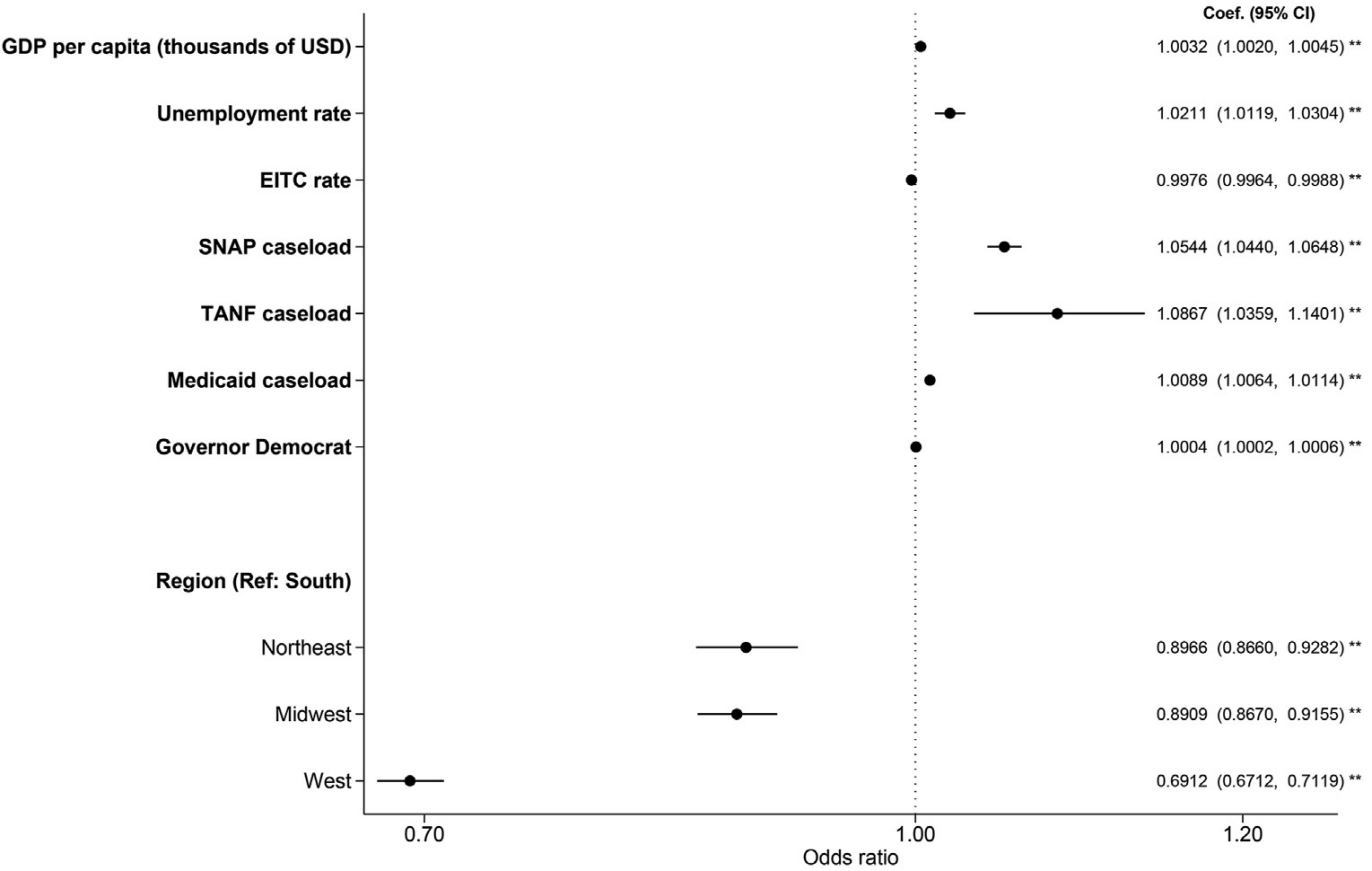
State-level predictors of WIC uptake. Sample (*N* = 288,531) was drawn from PRAMS participating sites from 2004 to 2019 and included respondents with live-born singleton births with a gestational age of 20–44 wk at delivery, who were eligible for WIC (based on income threshold or Medicaid receipt during pregnancy). Multivariable logistic regressions examined the association between each predictor and WIC uptake, controlling for individual- (age, race/Hispanic origin, education, language, marital status, prepregnancy diabetes, pregnancy intent, family size, and family income) and state-level covariates and the year of delivery. The filled circles and lines indicate ORs and 95% CIs, respectively. ∗∗*P* < 0.01. EITC, earned income tax credit; PRAMS, Pregnancy Risk Assessment Monitoring System; SNAP, Supplemental Nutrition Assistance Program; TANF, Temporary Assistance for Needy Families; WIC, Special Supplemental Nutrition Program for Women, Infants, and Children.

For state-level predictors (Figure 3), respondents living in states with a higher EITC rate and in the Northeast, Midwest, and West (compared with the South) had lower odds of WIC uptake during pregnancy. Higher state GDP per capita, higher unemployment rate, higher SNAP, TANF, and Medicaid caseloads, and Democrat governor were associated with higher odds of WIC uptake.

### Secondary analysis

In analyses stratified by time period, the association of WIC receipt with race/Hispanic origin, language, marital status, family size, and prepregnancy diabetes was strongest during the 2009–2015 period (TABLE 3, TABLE 4). The state GDP per capita was no longer significantly associated with uptake during the post-2016 period. The state unemployment rate was only significantly associated with uptake during the pre-2009 period. The state EITC rate was positively associated with uptake during the pre-2009 period, which was in the opposite direction as the main analysis and other time periods. The TANF caseload was associated with higher odds of WIC uptake during the pre-2009 period, whereas it was associated with lower odds of uptake during other time periods. Participants living in the Northeast had higher odds of WIC uptake during the post-2016 period, which was in the opposite direction of the main analysis.

**TABLE 3:** Individual-level predictors of WIC uptake by time period

Predictors	Time period		
	2004–2008	2009–2015	2016–2019
	OR (95% CI)	OR (95% CI)	OR (95% CI)
Age, y (reference: <25)			
<25–34	0.77^1^	0.79^1^	0.84^1^
	(0.75, 0.80)	(0.76, 0.82)	(0.81, 0.88)
>35	0.63^1^	0.66^1^	0.79^1^
	(0.60, 0.66)	(0.63, 0.69)	(0.75, 0.85)
Race/Hispanic origin (reference: White)			
Black	1.55^1^	2.06^1^	1.77^1^
	(1.48, 1.61)	(1.98, 2.15)	(1.69, 1.86)
Hispanic	1.58^1^	1.86^1^	1.59^1^
	(1.50, 1.67)	(1.77, 1.94)	(1.50, 1.69)
Asian/Pacific Islander	0.97	1.29^1^	1.25^1^
	(0.91, 1.03)	(1.22, 1.37)	(1.14, 1.37)
American Indian/Alaska Native	1.56^1^	2.10^1^	2.06^1^
	(1.45, 1.68)	(1.94, 2.27)	(1.90, 2.24)
Other	1.47^1^	1.45^1^	1.47^1^
	(1.32, 1.65)	(1.36, 1.55)	(1.36, 1.59)
Education (reference: <high school)			
High school	0.90^1^	0.86^1^	0.91^1^
	(0.86, 0.93)	(0.82, 0.90)	(0.86, 0.96)
More than HS	0.63^1^	0.61^1^	0.69^1^
	(0.61, 0.66)	(0.59, 0.64)	(0.65, 0.73)
English	0.74^1^	0.61^1^	0.79^1^
	(0.69, 0.80)	(0.57, 0.66)	(0.73, 0.86)
Married	0.72^1^	0.66^1^	0.74^1^
	(0.70, 0.75)	(0.64, 0.68)	(0.71, 0.77)
Prepregnancy diabetes	1.17^1^	1.39^1^	1.23^1^
	(1.07, 1.28)	(1.28, 1.51)	(1.12, 1.36)
Pregnancy intended	1.02	0.99	0.97
	(0.99, 1.05)	(0.96, 1.02)	(0.93, 1.01)
Family size	1.01^2^	1.05^1^	1.03^1^
	(1.00, 1.02)	(1.04, 1.06)	(1.02, 1.05)
Family income (thousands of USD)^3^	0.986^1^	0.9815^1^	0.9821^1^
	(0.9860, 0.9877)	(0.9806, 0.9823)	(0.9809, 0.9833)

Sample (*N* = 288,531) was drawn from PRAMS participating sites from 2004 to 2019 and included respondents with live-born singleton births with a gestational age of 20–44 wk at delivery, who were eligible for WIC (based on income threshold or Medicaid during pregnancy). Multivariable logistic regressions examined the association between each predictor and WIC uptake, controlling for individual-level covariates, state-level covariates (GDP per capita, unemployment rate, EITC rate, SNAP, TANF, and Medicaid caseloads, governor Democrat, and census region), and the year of delivery. For ease of interpretation, state-level covariates are presented separately in Table 4.

EITC, earned income tax credit; PRAMS, Pregnancy Risk Assessment Monitoring System; SNAP, Supplemental Nutrition Assistance Program; TANF, Temporary Assistance for Needy Families; WIC, Special Supplemental Nutrition Program for Women, Infants, and Children.

^1^*P* < 0.01.

^2^*P* < 0.05.

3 Inflation adjusted to 2018 US dollars based on the maximum income value in each income category.

**TABLE 4:** State-level predictors of WIC uptake by time period

State-level predictor	Time period		
	2004–2008	2009–2015	2016–2019
	OR (95% CI)	OR (95% CI)	OR (95% CI)
GDP per capita (thousands of USD)	1.006^1^	1.005^1^	0.9977
	(1.003, 1.008)	(1.003, 1.007)	(0.9952, 1.0002)
Unemployment rate	1.03^1^	0.997	0.98
	(1.01, 1.04)	(0.984, 1.010)	(0.95, 1.02)
EITC rate	1.006^1^	0.995^1^	0.995^1^
	(1.003, 1.008)	(0.994, 0.997)	(0.992, 0.997)
SNAP caseload	1.07^1^	1.09^1^	1.04^1^
	(1.05, 1.09)	(1.08, 1.11)	(1.02, 1.06)
TANF caseload	1.81^1^	0.89^1^	0.72^1^
	(1.65, 1.98)	(0.83, 0.97)	(0.66, 0.80)
Medicaid caseload	1.016^1^	1.002	1.010^1^
	(1.010, 1.023)	(0.998, 1.006)	(1.006, 1.015)
Governor Democrat	0.9998	1.0010^1^	1.0005^2^
	(0.9995, 1.0001)	(1.0006, 1.0013)	(1.0000, 1.0010)
Region (reference: South)			
Northeast	0.62^1^	0.92^1^	1.53^1^
	(0.59, 0.66)	(0.87, 0.97)	(1.42, 1.65)
Midwest	0.79^1^	0.94^1^	0.91^1^
	(0.75, 0.83)	(0.90, 0.99)	(0.86, 0.96)
West	0.72^1^	0.69^1^	0.81^1^
	(0.68, 0.76)	(0.65, 0.72)	(0.76, 0.87)

Sample (*N* = 288,531) was drawn from PRAMS participating sites from 2004 to 2019 and included respondents with live-born singleton births with a gestational age of 20–44 wk at delivery, who were eligible for WIC (based on income threshold or Medicaid during pregnancy). Multivariable logistic regressions examined the association between each predictor and WIC uptake, controlling for individual-level covariates, (age, race/Hispanic origin, education, language, marital status, prepregnancy diabetes, pregnancy intent, family size, and family income), state-level covariates, and the year of delivery. For ease of interpretation, individual-level covariates are presented separately in Table 3.

EITC, earned income tax credit; PRAMS, Pregnancy Risk Assessment Monitoring System; SNAP, Supplemental Nutrition Assistance Program; TANF, Temporary Assistance for Needy Families; WIC, Special Supplemental Nutrition Program for Women, Infants, and Children.

^1^*P* < 0.01.

^2^*P* < 0.05.

**FIGURE 4:**
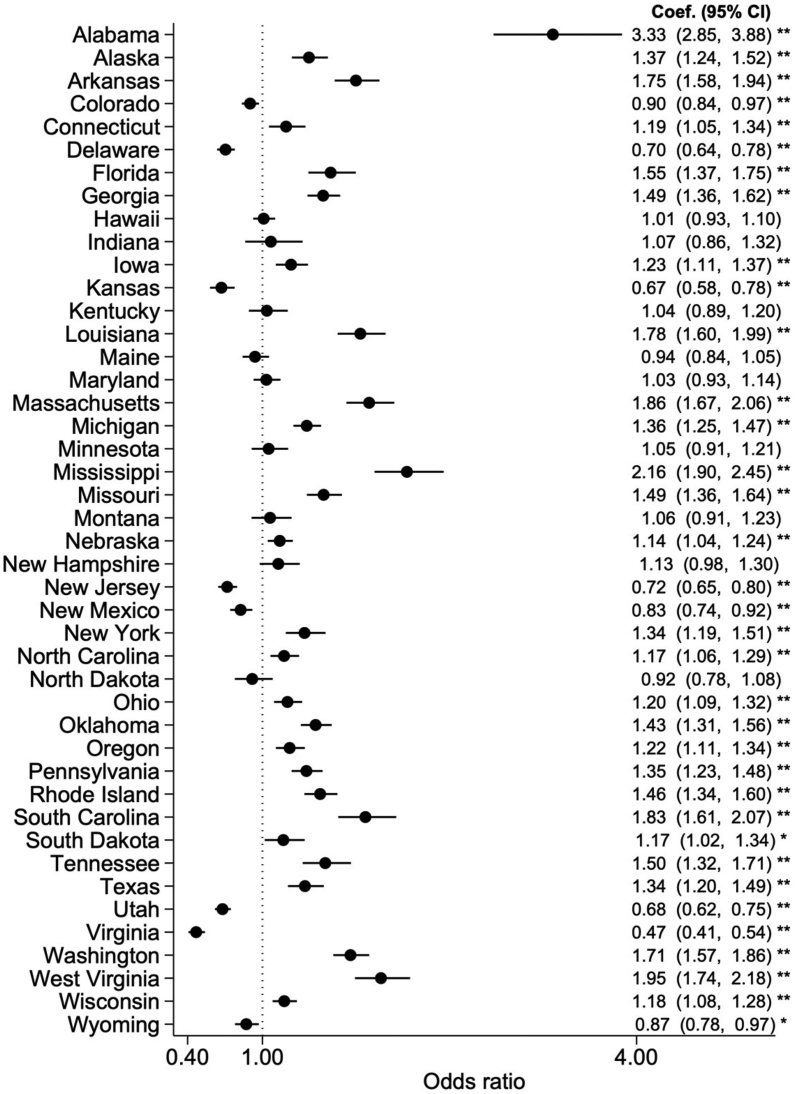
Variation in WIC uptake by state. Sample (*N* = 288,531) was drawn from PRAMS participating sites from 2004 to 2019 and included respondents with live-born singleton births with a gestational age of 20–44 wk at delivery, who were eligible for WIC (based on income threshold or Medicaid during pregnancy). The values presented here represent the coefficients on state indicator variables in multivariable logistic models also adjusting for individual-level covariates, state-level covariates, and the year of delivery. The reference state for this analysis was Illinois. Note that some states do not participate in PRAMS and are therefore not included in this analysis. The filled circles and lines indicate ORs and 95% CIs, respectively. ∗∗ *P* < 0.01, ∗ *P* < 0.05. PRAMS, Pregnancy Risk Assessment Monitoring System; WIC, Special Supplemental Nutrition Program for Women, Infants, and Children.

When state indicators were included, there was a range of variation in uptake, even after adjusting for other predictors (Figure 4). Twenty-seven states were associated with higher odds of WIC uptake compared with the reference state of Illinois, with Alabama having the strongest association (OR: 3.33; 95% CI: 2.85, 3.88). Eight states were associated with lower odds of WIC uptake, with Virginia having the strongest association (OR: 0.47; 95% CI: 0.41, 0.54). In sensitivity analyses imputing missing values, the results were similar to the main findings (Table 2).

## Discussion

This study provides some of the first contemporary evidence examining predictors of self-reported WIC uptake in a large national sample of WIC-eligible individuals. Initial bivariate results are similar to the recent USDA report, which draws from national administrative data that may be subject to less measurement error in the WIC variable itself; however, the USDA report does not contain information on eligible nonparticipants, does not include as many years of data or demographic variables, and focuses on bivariate analyses compared with the multivariable analyses conducted here [22]. Multivariable analysis showed that WIC-eligible racial/ethnic minority respondents had higher uptake than White respondents, whereas older respondents had lower rates of uptake. Younger pregnant individuals are more likely to be socioeconomically disadvantaged and perhaps in greater need of income and safety net support [23,24]; the results that showed that they have higher uptake indicate that the benefits are reaching this higher-risk group. Although most coefficients were significant statistically, for some variables (e.g., family income and governor’s party), the coefficients were relatively small and may not represent meaningful changes except at a population level [25].

The results suggest that eligible respondents with lower socioeconomic status are more likely to receive benefits. These results are in line with recent national and state reports showing higher WIC uptake among racial minority individuals and non-English speakers [14,26]. Previous work using national data prior to 2000 found that individuals who were Black or Hispanic were more likely to participate in WIC than non-Hispanic White individuals and also found low-income households, married individuals, and those with lower education levels were more likely to participate in WIC after adjusting for individual-level characteristics [27]. Recent work using New York City’s 2004–2007 PRAMS data found that Hispanic and Black respondents were more likely to participate, as were those with unintended pregnancies and with less social support [28]. The current study expands on the findings of prior work by using more recent years and a larger, national sample.

State-level factors were associated with WIC uptake, even after adjusting for individual-level factors. Individuals are adjunctly eligible for WIC if they are enrolled in Medicaid, SNAP, or TANF; therefore, the higher uptake associated with higher caseload of these programs could indicate that these states have decreased administrative barriers and more information on WIC benefits for their enrollees. Prior work found that adjunctive eligibility for WIC through SNAP was associated with higher WIC uptake, highlighting the importance of interagency administration and outreach [27]. Additionally, 80% of the WIC-eligible individuals reported participation in Medicaid, SNAP, or TANF in 2018, making communication about WIC benefits an important pathway for increasing participation [29]. Earlier studies found that states with higher enrollment in federal assistance programs, such as Medicaid, SNAP, or TANF, were associated with higher WIC participation [27,30]. The variation in uptake across states after accounting for state-level characteristics, as evidenced by coefficients on state indicator variables in the models, may be due to variations in how state WIC programs are administered. A recent study found that states that transitioned from a WIC paper voucher system to electronic benefits transfer saw a 7% increase in uptake among pregnant individuals [31]. The unemployment rate, Medicaid expansion, and stricter WIC eligibility rules have been associated with lower uptake, whereas the transition of the Aid to Families with Dependent Children (AFDC) program to the Temporary Assistance for Needy Families (TANF) program was associated with increased uptake [27,30]. However, these studies have relied on small samples from a single state or used historical data that may not be generalizable in the present day.

The association between predictors and WIC uptake was strongest during the 2009–2015 period compared to other periods. This period includes the 2009 WIC food package revision, which included improvements to the nutritional quality of the food package and expanded cultural food options [32]. Recent work shows that the 2009 revision has led to increased demand and sale of healthy foods and has increased the availability, variety, quality, and prices of foods in retail stores, especially in low-income areas and in predominantly Black census tracts [[33], [34], [35], [36]]. The stronger association between individual factors during this period may be due in part to the increased availability of culturally appropriate healthy food options in the revised package and more access to WIC-approved foods in low-income neighborhoods. The recent increase in WIC participation post–COVID-19 pandemic (which was not a focus of this study) may have been due to waivers allowing visits and benefits to be issued remotely [18]. Future work should examine other factors associated with increased participation during and after the pandemic.

The study estimates of WIC uptake among eligible pregnant respondents (75%) were higher than the USDA estimates (52% in 2019) [14]. This is in part because the study sample excluded some individuals at the higher end of the income eligibility range, given PRAMS data limitations on self-reported income. Self-reported safety net benefit receipt may also be unreliable [37], and people may not have reported the same family size and income to PRAMS as they did to their local WIC offices to determine eligibility.

This study has several strengths. It includes more years and more recent data relative to prior work, providing a more contemporary and longitudinal picture of predictors of WIC participation. It also includes a large number of respondents across multiple states, making results more generalizable than previous studies. The granularity of PRAMS data also allows for better classification of WIC eligibility and richer covariate predictors.

There are also limitations to this study. The first limitation was the use of a categorical self-reported income variable to determine WIC eligibility, which may have contributed to misclassification. Relatedly, the WIC uptake was also self-reported, although there is a high degree of agreement between these self-reported measures in PRAMS and birth certificates [38]. The linkage of administrative data on income and safety net participation with self-reported demographics is challenging in the United States context, and this can be a focus of future work. Information on other factors that could affect WIC participation such as knowledge about WIC, challenges in uptake, and stigma about program participation were not available in PRAMS, which should be addressed through future qualitative and survey research. Although this study included state-level data on EITC, SNAP, and TANF, individual-level receipt or enrollment in these programs was not available in the current dataset. Given the findings documenting an association between these state-level safety net programs and WIC uptake, future studies might explore these predictors at the individual-level. Furthermore, although certain state-level predictors such as governor party affiliation were statistically significant, they do not represent feasible targets for policy change. Additionally, most of the associations that we observed between state-level predictors were small in magnitude and may not reflect substantial impacts. Finally, some of the predictors included in the models are correlated with one another, and these analyses are correlational rather than causal; hence, the findings for any single variable should be interpreted with caution.

Given the decline in WIC uptake in recent years, this study contributes important new information on the factors that are associated with WIC uptake to inform appropriate interventions to improve participation. Results show that several individual-level characteristics correlated with higher socioeconomic status (albeit among a group of already low-income respondents) are associated with lower WIC uptake, and these findings can help in the tailoring of outreach and communications to improve WIC uptake. States with higher safety net program participation also have higher WIC uptake, making it important to consider how policy changes that affect other safety net programs will affect WIC uptake and suggesting possible synergies in terms of streamlining application processes for individuals eligible for multiple programs.

## Funding

This publication was supported by the National Center for Advancing Translational Sciences, National Institutes of Health, through UCSF-CTSI grant (UL1 TR001872). The contents of this publication are solely the responsibility of the authors and do not necessarily represent the official views of the NIH.

## Author contributions

The authors’ responsibilities were as follows – DC, AG: analyzed the data; DC, RH: wrote the first draft of the paper; RH: designed the research; and all authors: edited the manuscript substantively for critical content and read and approved the final manuscript.

## Conflict of interest

The authors report no conflicts of interest relevant to this article.

## Data availability

The data described in the manuscript and a codebook are available upon request from the Pregnancy Risk Assessment Monitoring System (https://www.cdc.gov/prams/prams-data/researchers.htm). The analytic code will be available upon request from the authors.

## Appendix A Supplementary data

Multimedia component1 Multimedia component1
