# Supplementary material for: Predictors of WIC uptake among low-income pregnant individuals: a longitudinal nationwide analysis
Source: Am J Clin Nutr. 2023 Apr 23;117(6):1331–41. doi: 10.1016/j.ajcnut.2023.04.023 (PMC10447486; doi:10.1016/j.ajcnut.2023.04.023)
Supplement: Multimedia component1 [file mmc1.pdf]

SUPPLEMENTARY MATERIAL

Supplementary Methods

Additional Detail on Sample Selection

The state of Vermont does not release variables for race/Hispanic-origin and was not included in our analysis, since this was considered a key predictor variable.

Additional Detail on Explanatory Variables

For the language variable, PRAMS does not consistently include a variable for language spoken at home, so this was operationalized as whether the survey was conducted in English or Spanish. Furthermore, we coded language as English if language for survey was missing. Of note, starting in 2009, PRAMS also included Chinese as a survey language, but due to the small number of observations (<1%) these were dropped from our analyses.

Measures of state caseloads of other social programs—including Supplemental Nutrition Assistance Program, Temporary Assistance for Needy Families, and Medicaid—were calculated as the population-weighted caseload of each service (i.e., caseload/population×100). State-level characteristics were obtained from the University of Kentucky Center for Poverty Research and were merged with individual-level data based on participant's state of residence and the year prior to the survey year.(1)

Additional Sensitivity Analysis

We conducted additional sensitivity analyses to examine the robustness of our results. First, given that the number of states that meet PRAMS criteria for data release changes every year, we

conducted a sensitivity analysis in which we restricted the data set to states with no more than three years of missing data during our study period. This ensured that year-to-year variation in the composition of the sample did not bias estimates. Results were similar to our main analysis in both magnitude and direction (results available upon request). Lastly, individual-level predictors varied in missingness from 1% for age to 8% for income. We therefore repeated the analysis using multiple imputation using chained equations (10 imputations).(2) Results were similar to our main findings (Table 2).

Supplementary References

1. University of Kentucky Center for Poverty Research. National Welfare Data. In: University of Kentucky Center for Poverty Research; 2020.
2. White IR, Royston P, Wood AM. Multiple imputation using chained equations: Issues and guidance for practice. Statistics in Medicine 2011;30(4):377-399.

Supplementary Table. States Included in Analysis

1. Alabama
2. Alaska
3. Arkansas
4. Colorado
5. Connecticut
6. Delaware
7. Florida
8. Georgia
9. Hawaii
10. Illinois
11. Indiana
12. Iowa
13. Kansas
14. Kentucky
15. Louisiana
16. Maine
17. Maryland
18. Massachusetts
19. Michigan
20. Minnesota
21. Mississippi
22. Missouri
23. Montana
24. Nebraska
25. New Hampshire
26. New Jersey
27. New Mexico
28. New York[a]
29. North Carolina
30. North Dakota
31. Ohio
32. Oklahoma
33. Oregon
34. Pennsylvania
35. Rhode Island
36. South Carolina
37. South Dakota
38. Tennessee
39. Texas
40. Utah
41. Virginia
42. Washington
43. West Virginia
44. Wisconsin
45. Wyoming

[a] Although the Pregnancy Risk Assessment Monitoring System administers surveys separately for New York State and New York City, responses were aggregated into one group for the purpose of this analysis.
